# Pay talk in contemporary workplaces

**DOI:** 10.1093/sf/soae130

**Published:** 2024-09-09

**Authors:** Patrick Denice, Jake Rosenfeld, Shengwei Sun

**Affiliations:** Department of Sociology, University of Western Ontario, London, ON N6A 5C2, Canada; Department of Sociology, Washington University in St. Louis, St. Louis, MO 63130, United States; National Women’s Law Center, Washington, DC 20005, United States

**Keywords:** inequality/social stratification, work/labor process, organizations/management

## Abstract

Drawing on a unique survey of US workers with information about their employers’ policies on pay discussions and whether workers engage in such talk with their coworkers, we provide the most comprehensive investigation into pay talk in workplaces to date. Unlike existing treatments, we focus on core organizational and relational factors that influence whether workers talk about pay. We theorize pay talk as a challenge to managerial discretion, and we hypothesize that organizational attributes related to pay-setting influence workers’ willingness to discuss wages and salaries with colleagues. Managers, in turn, combat such challenges to their discretion by instituting pay secrecy rules. Particular relational factors within organizations are related to workers’ violations of these rules. Findings indicate that the likelihood of pay discussions varies by workplace pay secrecy rules, managerial relations within organizations, and, in certain model specifications, sector and career turning points. Among status characteristics, only age is associated with discussing pay, with younger workers significantly more likely to talk about pay and to violate organizational rules meant to suppress pay discussions.

## Introduction

“…money—along with sex, politics, and religion—is a topic best avoided in polite conversation…”.

—Tim Herrara, *New York Times*, August 31, 2018

In just the last decade, growing demands for pay information have motivated over a dozen states to enact laws sanctioning employers who ban worker discussions of wages and salaries.^1^ Journalistic accounts suggest that the reluctance to discuss pay is eroding among younger workers (e.g., Fox 2022); yet, characteristics beyond age that correlate with the likelihood of talking about pay remain unknown. In this article, we investigate the key organizational and relational factors shaping whether individuals discuss pay with their co-workers. Drawing on a unique nationally representative survey, we provide the most comprehensive analysis of pay talk among workers to date.

Our article proceeds in two parts. First, we move beyond existing treatments that emphasize the importance of individual status characteristics to focus on organizational factors that help determine whether workers discuss pay. Theoretically, we conceptualize pay talk as a possible challenge to managerial discretion. The likelihood that a worker will challenge management in this way rests on organizational attributes related to pay-setting. In particular, workers will be more likely to talk about pay in organizations where pay information is powerful, that is, where the information revealed is relevant to bargaining over the distribution of organizational revenue. This relevance depends on two organizational features: whether pay information is a scarce resource (i.e., not everyone has access to it), and whether managers have significant discretion over pay-setting.

In these organizations where information about pay is unequally distributed and pay-setting power rests with management, revelations about pay disparities among otherwise similar workers can have a leveling effect. Research finds that workers perceive horizontal inequities in pay as unfair (Levine 1993), and that maintaining large pay differentials among otherwise similar workers can result in morale issues if pay secrecy is breached (Akerlof and Yellen 1990; Card et al. 2012). Managers who desire the widest discretion in pay-setting therefore have an incentive to close off the dissemination of this particular network resource. They do so by implementing and maintaining pay secrecy rules, which refer to organizational rules that prohibit or discourage workers from talking about wages or salaries (Gely and Bierman 2003). The implementation of these rules “is an attempt by employers to increase their power during wage negotiations” (Sauer et al. 2021, p. 955) by reducing workers’ access to potentially powerful information about pay.

Theoretically, we conceive of a pay secrecy rule as a *rule of managerial discretion*. Organizational research has focused on how workplace rules and regulations impinge on managerial discretion, whether they be diversity policies (Kalev, Dobbin, and Kelly 2006), formal grievance procedures (Chamberlain et al. 2008), or performance evaluations (Castilla 2011a). Other work reveals how managers bypass such rules and regulations, or invoke them selectively, to claim back autonomy in decision-making and augment dominant actors’ power (Roscigno 2007, 2011; Castilla 2011b). Rules of managerial discretion, conversely, allow for greater managerial autonomy, and their violation constrains managerial prerogatives. They are bureaucratic structures that widen discretion, remove constraints on powerful actors, and allow for particularistic treatment of subordinates. Their infringement serves, paradoxically, as a limiting mechanism on biased or otherwise unfair treatment in organizations.

Having established that these rules are an important organizational feature structuring pay talk in the first part of investigation, in the second, we turn to the relational factors associated with workers’ likelihood of breaking them. As Martin et al. (2013, p. 551) emphasize, “routine rule-breaking is ubiquitous” within organizations, and we contend that a set of relational processes within the walls of the organization structure workers’ violations of pay secrecy rules. These violations are relational by definition in that they require multiple people. And whereas the motivations behind many other types of workplace rule-breaking are “myriad and idiosyncratic,” (p. 555) we argue that workers often breach pay secrecy policies to level power differentials within organizations, shifting authority downward. Thus, we hypothesize that workers are more likely to do so when the revealed information would have an impact on the distribution of organization rewards—when the perceived payoff is greatest. These are the situations in which violating a rule of managerial discretion can constrain managerial action.

In addition to contributing to the literature on workplace rule-breaking, especially research that focuses on the organizational and relational underpinnings of workplace deviance (e.g., Hibel and Penn 2020), our two-part investigation adds to the emerging work on the power of transparency. Prior research establishes that access to vital information within organizations can empower workers, whether through higher pay (Kleiner and Bouillon 1988, 1991; Rosenfeld and Denice 2015) or reduced stress on the job (Zheng et al. 2021). And whereas this past work focuses on managers as the core actors determining whether workers have access to key information, our investigation centers on worker behavior and the organizational and interactional factors that help structure it. A growing number of high-profile anecdotal cases reveal the serious potential costs of pay secrecy (Timm 2014; Kaplan 2015). Emerging academic evidence suggests greater pay transparency may translate to material benefits for women workers (Kim 2015) and overall reductions in gender pay disparities (see Bennedsen, Larsen and Wei 2023 for a recent survey of the literature). Other research suggests managers bargain harder in pay negotiations in contexts of relative transparency (Cullen and Pakzad-Hurson 2023). With the exception of Devaraj and Patel (2022), what unites these investigations is the finding that transparency affects the distribution of available revenue and therefore has the power to change pay-setting dynamics within organizations. Yet, the organizational and relational factors associated with workers’ desire for and willingness to obtain pay information remain unexplored.

Our investigation also informs ongoing policy debates over pay secrecy rules in workplaces. The emerging set of state laws ratcheting up penalties on employers who maintain pay secrecy rules aims to level existing pay disparities, especially between men and women. However, these laws do not mandate transparency; they simply allow workers to discuss wages and salaries without fear of employer retaliation. Our data indicate that fewer than half of workers talk about pay even in those workplaces where pay discussions are allowed. Policies aimed to facilitate the spread of pay information may prove ineffective if workers remain reluctant to ask about the pay of their peers. In other words, most workers may not need a workplace rule precluding salary discussions, given the discomfort they feel talking about pay more generally (Soled 2022, p. 970). Lawmakers implicitly assume that workplace pay secrecy rules are uniformly effective in staunching the flow of pay information. They also assume that the absence of such a rule will prove effective at spreading pay information and empowering disadvantaged workers. Our survey is unique in being the first nationally representative one that includes both information about US workers’ knowledge of and willingness to discuss co-workers’ pay and indicators of pay secrecy rules in workplaces.

To foreshadow key findings from the first part of our investigation, we find general support for our claim that discussing pay can challenge managerial discretion. We contend that sector and the presence or absence of a pay secrecy rule comprise two organizational features that proxy for the scarcity of pay information and managerial control over pay-setting. Both are significantly associated with workers’ likelihood of discussing pay with colleagues, although the significance of sector is sensitive to sample restrictions. Contrary to expectations, our third proxy—union membership—is not.

The second part of our analysis investigates whether our conceptual framing of pay secrecy rules as rules of managerial discretion has empirical backing. We examine key relational factors hypothesized to affect the likelihood and timing of rule breaches. The impetus to break a rule of discretion should be highest when relationships with one’s managers are poor, and management is not seen as a trustworthy partner in the pay-setting process. A lack of trust in one’s superiors likely translates to a lack of trust in pay-setting, and a greater motivation to arm oneself with potentially powerful information for relational claims-making over organizational revenue (Tomaskovic-Devey and Avent-Holt 2019). In this way, violations of pay secrecy rules can activate “silenced” claims (164) by serving as a preliminary step in claims-making over organizational resources.^2^ And the timing of such violations should coincide with pay negotiations. Presumably, these are the periods in which the revealed information could shift bargaining dynamics in workers’ favor. Our analyses and numerous robustness checks establish that managerial relations are consistently associated with the violation of pay secrecy rules. Our second relational factor—whether the worker plans to ask for a raise—is positively associated with breaking pay secrecy rules, although the significance of the effect varies by model.

## Pay talk and rules of managerial discretion

That discussing pay with anyone, let alone with colleagues, challenges a long-standing, still-powerful taboo is a cliché frequently invoked in academic and non-academic arenas. Existing accounts largely assume its presence and power; few studies attempt to measure it or document its various dimensions (for rare exceptions, see Cullen and Perez-Truglia 2022, 2023). Cullen and Perez-Truglia (2023, p. 1) define the salary taboo as the “…norm around salary privacy that discourages coworkers from revealing or asking others about salary information.” This definition constrains the reach of the taboo to those situations in which wages and salaries are private. This condition is not universal, as in workplaces that publicize their pay scales, and thus the reach of the taboo will vary. The hesitancy to share or ask about otherwise concealed pay information is widespread. In their recent case study, Cullen and Perez-Truglia (2023, p. 10) find that over two-thirds of workers thought asking colleagues about their pay was socially unacceptable; nearly nine in ten said they would feel uncomfortable asking for the information.

We view the reluctance to talk about pay not as a fixed individual characteristic, or a universal cultural norm, but as an ongoing construct generated from a set of organizational and relational factors (Tomaskovic-Devey and Avent-Holt 2019, p. 191). Press accounts suggest this reluctance is eroding among younger workers (Weller 2018), and policy reports indicate the taboo’s hold may be weaker among both younger generations and women (Sun, Rosenfeld, and Denice 2021). While we do not deny the potential importance of status characteristics—especially age—on workers’ willingness to discuss pay, we expand beyond the usual focus on individual characteristics by identifying the core set of organizational and relational factors likely to influence whether workers challenge the cultural prescription against talking about pay.

Maintenance of salary secrecy can serve managers’ interests by simultaneously widening their discretion over pay-setting while narrowing the available resources workers have when negotiating pay. Managers who are confident that their subordinates are unaware of organizational pay scales can take advantage of this information asymmetry in relational claims-making over organizational revenue. Organizational features affect employee behavior of all sorts (Martin et al. 2013, p. 552), and different features—such as those related to wage bargaining—should influence a worker’s willingness to discuss pay, thereby allowing them to gain potentially powerful information.

Powerful actors within organizations that keep pay secret thus have an incentive to mobilize the cultural taboo against discussing pay (Tomaskovic-Devey and Avent-Holt 2019, p. 191). They do so in one primary way: instituting pay secrecy rules. Pay secrecy rules are widespread and unevenly patterned across workplaces, with research finding that organizational factors such as sector and union presence are key correlates of their presence or absence (Rosenfeld 2017). In private sector workplaces without a union presence, pay secrecy policies are the norm (Rosenfeld 2017, fig. 3). These rules range from informal workplace proscriptions on discussing pay to formal rules codified in employee handbooks with penalties specified for violators. Regardless of their form, the impetus behind them is to maintain information asymmetries between workers and their superiors, widening the discretion pay-setters have when allocating organizational revenue.

We argue that pay secrecy policies are workplace rules granting greater managerial discretion. These are workplace rules that enlarge rather than constrain employers’ scope of action. Organizational scholarship has evolved past prior notions of bureaucratic procedures as necessarily constraining, with newer scholarship highlighting the ubiquity of workplace rule-breaking (Martin et al. 2013). Other research (Roscigno 2011) highlights how powerful actors selectively invoke workplace rules and procedures to act in self-interested, sometimes malicious manners—in other words, to widen the space in which they can act toward subordinates. We maintain that these rules of managerial discretion themselves accomplish this task. There is no reason for an employer interested in maintaining the greatest scope over setting pay at her organization to invoke a pay secrecy rule selectively. The rule itself grants the pay-setter such freedom in claims-making over organizational revenue. In this way, pay secrecy rules are similar to other common bureaucratic structures such as non-compete clauses in employment contracts (Starr, Prescott, and Bishara 2021). These also grant employers greater freedom to act and power over subordinates by reducing workers’ exit options (Marx 2011). What marks a rule of managerial discretion is that it represents a layer of bureaucracy that removes constraints on powerful actors while imposing limitations on subordinates.

## Core hypotheses

### Organizational factors and pay talk

A core contention of this investigation is that the willingness or reluctance to share pay information is neither a universal norm nor an individual-level phenomenon, but is shaped according to a set of organizational features. Those that pertain to the scarcity and relevance of pay information should be especially important in influencing whether or not a worker will discuss pay. This is due to the potential power pay information can have in bargaining, which depends on whether pay information is widespread and whether managers have significant control over pay-setting.

Three features in particular proxy for the availability of pay information and the pay-setting dynamics within an organization. The first is sector. Public sector organizations are much more likely to publicize organizational pay scales compared with private sector enterprises. Our data indicate that nearly three-quarters of public sector employees say their organization publicizes pay compared with just over a tenth of employees of private-sector, for-profit establishments. Also, public sector organizations often feature highly centralized compensation procedures, limiting the role of individual managers in setting pay and individual workers in bargaining for raises (for recent discussions, see Mandel and Semyonov 2021; Perera and King 2021, p. 183; Sauer et al. 2021, p. 939). Given that a primary motivation behind discussing pay is to discover potentially powerful information in relational claims-making, we hypothesize that public sector workers are less likely talk about pay (*Hypothesis 1*) since so many are already privy to pay information, and any information revealed is unlikely to have much relevance to pay-setting.

Our second potentially important organizational feature is unionization. Aside from offering general protection against managerial overreach, as well as standardized grievance procedures for handling workplace conflicts, unions have fought for greater transparency about organizational finances (Rosenfeld and Denice 2015, p. 1046). Existing research finds that unionized organizations are significantly less likely to have a pay secrecy policy in place (Rosenfeld 2017), and our data indicate that over two-thirds of unionized respondents report that pay scales are public information at their workplace. Even in those unionized establishments where pay information is not publicly provided, collective bargaining agreements restrict managerial flexibility in pay-setting and workers’ freedom to negotiate individual raises (Freeman and Medoff 1984, pp. 86–87; Sauer et al. 2021, p. 939). For these reasons, we hypothesize that unionized workers have less incentive to discuss pay compared with nonunion workers (*Hypothesis 2*).

Our final organizational factor we hypothesize affects pay talk is the presence or absence of a pay secrecy rule. We contend that in organizations where pay information is not public, workers subject to a pay secrecy rule are less likely to discuss pay with colleagues compared with workers in organizations without such speech restrictions. While this contention may seem obvious, popular discussions of the salary taboo often assume its universality (Wong 2018) or emphasize demographic factors—usually age—as the core source of variation in whether someone violates the taboo (Fox 2022). The organizational rules that potentially structure pay talk have remained largely unexplored. And a recent exception highlights the potential limitations of pay secrecy rules. In Cullen and Perez-Truglia’s case study (Cullen and Perez-Truglia 2022, p. 776), the authors find that despite the company’s informal policy against discussing pay, over half the workers surveyed discuss their salaries with co-workers at least once a year. Norm-breaking, at least in this firm, was fairly common.

Yet, given the congruence between the generalized cultural frame against discussing pay and workplace rules that enforce it (Tomaskovic-Devey and Avent-Holt 2019, p. 191), we expect organizational pay secrecy policies to have a dampening effect on workplace discussions about wages and salaries. Our data allow us to distinguish “informal” from “formal” secrecy rules, with the former referring to discouragement of pay talk by managers, and the latter referring to codified rules attached to specific punishments for violations. Workers in those organizations that “amplify” (Roscigno 2011) the common cultural frame upholding pay secrecy by instituting formal pay secrecy policies that contain the threat of punishment should have the lowest likelihood of talking about pay. In sum, we hypothesize that, in organizations where pay information is not public, workers subject to any pay secrecy policy are less likely to discuss pay (*Hypothesis 3a*) and those subject to a *formal* pay secrecy policy are the least likely (*Hypothesis 3b*).

### Relational factors and rules of managerial discretion

We view the violation of pay-secrecy rules as a relational process often aimed to augment worker power vis-à-vis managers in another relational process: pay-setting (Avent-Holt and Tomaskovic-Devey 2014; Rosenfeld and Denice 2015). Not all workers in all situations risk sanctions by breaking rules against discussing pay. Just as certain organizational features—most notably, pay secrecy rules—condition whether workers will discuss pay, within those organizations with such rules, certain relational factors likely structure the probability and timing of rule breaches.

First, poor managerial relations may encourage workers to violate managerial rules of discretion in order to obtain pay information. Lack of trust that management is bargaining in good faith raises workers’ incentive to arm themselves with such potentially powerful and scarce information. Additionally, past research has identified poor relations as important factors leading to workplace rule-breaking in general. As Peterson (2002, p. 49) notes, “perceptions of unfair treatment...can contribute to workplace deviance”, a finding corroborated by other research that emphasizes how workers’ feelings of injustice on the job lead to resistance of managerial authority (Roscigno and Hodson 2004). More recent research (Sauer et al. 2021, p. 950) finds that workers dissatisfied with their jobs are more likely to negotiate for higher pay. If violating pay secrecy rules is often a preliminary step to increase leverage in claims-making over organizational revenue, then such violations should be more common in contexts of poor managerial relations (*Hypothesis 4*).

Second, we define a worker’s likelihood of asking for a raise as a relational process involving a worker and her superior. Similar to Luekemann and Abendroth’s (2018) conception of workers’ discussions about career advancement with superiors as claims-making, we see a worker’s request for a raise as a claims-making moment meant to secure a greater share of organizational revenue.^3^ Workers armed with powerful information—whether it be information about organizational finances (Kleiner and Bouillon 1988, 1991; Rosenfeld and Denice 2015) or pay (Kim 2015)—enter negotiations with resources that could increase their likelihood of success. Lacking knowledge of a workplace’s finances or pay scales tilts negotiations in pay-setting toward employers, who can respond to requests with variations of “we cannot afford that” or “you are fairly compensated for your position.” We hypothesize that workers who plan to ask for a raise are more likely to violate pay secrecy rules (*Hypothesis 5*). If pay information is a powerful constraint on managerial prerogatives when it comes to setting pay, then workers who break this rule of managerial discretion should be more willing to exercise the power that comes with greater transparency, providing evidence that pay information is not neutral, or simply idle gossip, but can be a resource in relational claims-making over organizational revenue by activating previously silenced claims.

Recent research further substantiates this hypothesis, finding that workers’ desire to know their co-workers’ wages and salaries is highest at career turning points, such as when the worker is scheduled for a contract negotiation or a promotion (Cullen and Perez-Truglia 2023, p. 16). These are presumably the junctures when such information would be the most powerful in claims-making within organizations. As a result, relational processes may help structure not only the motivation but the timing of this particular type of workplace deviance.

## Data and methods

We partnered with the survey research firm GfK Knowledge Networks (now Ipsos) and fielded a national survey of US workers in the fall of 2017, and followed up with a subsample survey in late fall 2018. We restricted our survey to full-time employees aged 18 and over who are not self-employed. Respondents who met our study’s criteria were randomly sampled from GfK’s nationally representative panel. GfK selects respondents into their panel via address-based sampling. Once they are a part of the panel, respondents receive emails with links to the surveys for which they have been selected. GfK provides households without an internet connection with a web-enabled device and free internet service. We obtained 2,568 complete surveys in 2017, with a survey response rate of 59.6 percent. GfK followed up with 2,264 of our original respondents, and received 1,988 completed questionnaires for a follow-up response rate of 87.8 percent.^4^ Of those who completed the follow-up survey, 1,694 met our study’s parameters and are included in the full dataset of 4,262 person-years. We exclude an additional 33 individuals who are missing on our outcome variable (whether a respondent talks to colleagues about pay), for a final total sample size of 4,229 (consisting of 2,553 respondents from round 1 of the survey plus 1,676 of those same respondents who were re-surveyed in round 2). Sample weights produce estimates representative of the adult (18 and over) US population who are full-time employees.

Our survey includes a battery of items about wage and salary information dissemination in the workplace. Specifically, the survey asks: “Do you know the wages or salary levels of at least some of your co-workers?” For respondents who answer “yes”, the survey follows up with the question: “How do you know the wages or salary levels of at least some of your co-workers?” Options include “talking with co-workers” (our measure of pay talk), as well as “pay information publicly available”, “external website”, “your job title gives you access to pay information”, and “other reason”.

Table 1 shows whether respondents in our core analytic sample (*N* observations = 4,229) know about their coworkers’ pay and how they came by this information. Overall, 64 percent know the wages or salaries of at least some of their co-workers.^5^ And of those with such information, over half (56 percent) obtained it by talking with co-workers. Another 27 percent of respondents with pay information received it as part of their job duties, and just 6 percent acquired information about pay from online sources.

**Table 1:** Workers’ knowledge of their coworkers’ wages or salaries.

	Percentage
Know the pay of at least some of their co-workers	63.76
Of those who know their coworkers’ pay:	
From talking with coworkers	55.86
As part of their job duties	27.45
From online sources	6.43
Because pay information is made publicly available	25.22

*Note:* Table displays percentage of workers in our full pooled sample (*N* = 4229) who know the wages or salaries of at least some of their co-workers, and among those workers who know (*N* = 2692), how they obtained such information. Estimates are weighted by sample weights.

Knowledge can also come directly from workplaces that make wages and salaries publicly available. Our survey replicates an item from an Institute for Women’s Policy Research (IWPR) report that asked respondents in 2010 about their employer’s stance toward discussing wages and salaries in the workplace (Hayes and Hartmann 2011). Respondents could choose whether their employer publicized pay information, allowed for discussions about pay, discouraged discussions about pay, or had a formal workplace rule that sanctioned those who discussed pay.

To test Hypotheses 1–3, we conduct a series of logistic regressions, with respondents’ observations pooled over both waves of data collection and clustered standard errors to account for the autocorrelation in the error terms given respondents’ repeated observations. Pooled regression models make use of both between- and within-individual variation to estimate the relationships between a worker’s likelihood of talking about pay with their colleagues and our key independent variables of interest. For example, they help address the question of whether individuals are more or less likely to talk when they work under pay secrecy policies. Given the panel nature of our data, an alternative would be to include individual fixed effects. However, fixed effects models use only information from “switchers”—that is, those who have experienced any changes in the binary outcome of talking about pay (Kim and Do 2013). Such a limitation would substantially reduce the external validity of our results. Additionally, because most respondents did not change jobs or employers between the two rounds of data collection, both our outcome measure (engaging in pay talk) and our main independent variables of interest (including pay secrecy policy, union presence, and sector) are largely constant within individuals over time. Recent examples of a similar approach include Tuppat and Gerhards (2021) and Eesley (2016). That said, given the lack of substantial variation in our key variables across survey waves, as a robustness check we replicate our models using individuals’ responses from Round 1 of our survey only and present the results alongside our main models.

Our outcome variable is a dichotomous indicator of whether the respondent discusses pay with co-workers. We present two sets of model estimates using our pooled sample and Round 1 sample. The first set of models includes all of our core covariates, and the second includes these same controls but is limited to respondents not granted access to pay information as part of their jobs. This sample restriction provides the cleanest test of our hypotheses concerning pay talk, as the model is limited largely to those who must ask others in order to access pay information.

Our sector measure is a 3-category variable: public, private for-profit, and non-profit. For union status, models include a variable capturing union membership; supplemental models that replace individual membership with union presence at the respondent’s organization produce similar results. To test the effect of the organization’s approach to pay discussions on the likelihood workers discuss pay, we include a four-category item: pay is publicly provided, pay discussions are allowed, pay discussions are discouraged, or pay discussions are formally prohibited.

Given the paucity of prior empirical work exploring predictors of pay talk, we have little guidance to choose an appropriate set of core controls. As an initial strategy we proceed expansively, including a range of demographic, occupational, and workplace-level covariates that might plausibly influence a worker’s likelihood of discussing pay. We eventually narrow the set of covariates from these saturated models down to those that are either consistently associated with discussing pay with colleagues, theoretically presumed to influence pay discussions, or both. Foremost among these likely important controls is age. A growing chorus of journalists have suggested that the pay secrecy norm is eroding among younger workers (e.g., Dominus 2020; Gee 2017; Williams 2008). Emerging evidence buttresses the claim. A 2017 Bankrate survey of approximately 1,000 US adults found large generational differences in the percentage of respondents who had shared salary information with co-workers. Nearly a third of 18–36-year-olds reported that they had discussed pay with colleagues compared with less than 10 percent of workers between the ages of 53 and 71 (Berger 2017). We enter age into the models as a linear term. Including an age-squared term produces poorer model fit, while substituting age with generational indicators did not meaningfully improve the fit of the model. Aside from age, our main models also include measures of race/ethnicity (non-Hispanic white, non-Hispanic Black, Hispanic, and other) and sex.

Drawing on a strain of occupational research, we presume that occupational membership patterns pay talk. This research emphasizes white-collar employees’ devotion to their work (Blair-Loy 2005), and the professional and organizational processes that reward such devotion and punish those whose identity is not career-centered (Rao and Neely 2019; Reid 2015). Given widespread agreement among US workers that pay reflects individual performance (Rosenfeld 2021, p. 42), sharing information about pay reveals a core part of a worker’s identity, and can provide an indication of whether the worker is succeeding at her calling. The stakes of revealing pay are thus higher among workers in white-collar occupations whose identities are intertwined with their occupational membership. As a result, workers in white-collar and professional occupations may exhibit higher levels of adherence to salary secrecy.

To control for these possible occupational influences, main models include eleven major occupation dummies: (1) management/business/finance, (2) professional, (3) service, (4) sales, (5) office and administrative, (6) farming/fishing/forestry, (7) construction and extraction, (8) installation/maintenance/repair, (9) production, (10) transportation, and (11) missing. The first two categories comprise our “white collar/professional” occupations, presumed to display lower rates of pay discussions compared with such blue-collar occupations as construction, transportation, and production.

Main models also include the following set of organizational controls to lend confidence to our findings of theoretical interest: organization type (single establishment or one of several workplaces) and organizational size (<15, 15–49, 50–99, 100–499, 500+ employees). For workers at multi-establishment firms, organizational size refers to the number of workers at their particular workplace. We also include an indicator for employee tenure at an organization. The longer one works at an organization, the more likely a worker is to develop the bonds with colleagues often necessary to reveal and ask about sensitive information. Our employee tenure measure captures whether the worker has been at her organization for less than a year, 1 to less than 2 years, 2 to less than 5 years, 5 to less than 10 years, or at least a decade. Models estimated on the pooled sample also include an indicator for survey round.

To retain sufficient sample sizes, with the exception of our outcome variable, we include respondents missing on key covariates as a separate category. Missingness rates range from 0.3 percent (employee tenure) to 1.9 percent (organization’s approach to pay discussions).

The second part of our analysis focuses on the relational factors structuring violations of pay secrecy rules. For these models, the outcome variable remains the same—whether the worker discusses pay with colleagues—except now we limit the sample to workers subject to pay secrecy rules (*N* = 2037 for the pooled sample; *N* = 1207 for Round 1 survey responses only). For our main set of estimates, we model discussing pay with colleagues as a function of the core organizational predictors and controls from the first stage of the analysis, as well as our two relational factors of interest: a managerial relations scale and a dichotomous measure of whether the respondent plans to ask for a raise.

To create the managerial relations scale, we combine responses from six questions. The first three questions ask respondents to rate how good their managers are at seeking out employees’ views, responding to employees’ suggestions, and allowing employees to influence decisions (on a scale from 1 = very poor to 5 = very good). The second three questions ask employees to indicate whether managers deal with employees honestly, treat employees fairly, and keep their promises (on a scale from 1 = strongly disagree to 5 = strongly agree). The Cronbach’s alpha for this scale is .95 (factor loadings are shown in Table A1 of the appendix). We standardize the index so that the unweighted mean equals 0, with a SD of 1. Higher scores indicate more positive views of managers.

We include a final full model (Model 2) limited to workers who are not granted access to pay information as a part of their jobs. This is the portion of the sample that must violate these rules of managerial discretion in order to obtain potentially powerful pay information. By focusing on these workers in the second model, we provide the sharpest test of our hypotheses concerning the relational factors that influence whether a worker violates pay secrecy rules. As in the first stage of the analysis, pooled models in this second investigation include an indicator for survey round, and we cluster standard errors by respondent.

Table 2 presents key descriptives from our pooled sample of all respondents and our sample restricted to respondents subject to a pay secrecy policy at work, corresponding to the main models from both parts of our analyses.^6^ The first column represents the sample used to test Hypotheses 1–3. Among this sample, 36 percent of respondents report talking to their colleagues about pay. In terms of the organizational measures of theoretical interest, two-thirds of respondents work for private-sector, for-profit organizations, while just under a fifth work for the government. The percent unionized in our sample runs higher than the nation’s overall unionization rate, likely due to sample composition: ours is limited to full-time workers who are not self-employed. By definition, self-employed workers are ineligible for unionization and full-time workers are approximately twice as likely to belong to a union compared with part-time employees (see U.S. Bureau of Labor Statistics 2024). Overall, nearly half of respondents report that they are either discouraged or prohibited from talking about pay with co-workers, consistent with past estimates of the fraction of workers subject to these rules (Hayes and Hartmann 2011).

**Table 2:** Summary statistics.

	Full pooled sample	Restricted pooled sample
Talks with coworkers about pay	35.62	32.32
*Sector*		
Government	18.30	4.56
Private	67.32	82.57
Non-profit	12.97	11.72
Missing/refused	1.41	1.14
Union member	13.72	3.24
Missing/refused	1.15	0.62
*Pay secrecy policy*		
Information is public	24.54	
Discussion is permitted	25.12	
Discussion is discouraged	35.63	73.38
Discussion is prohibited	12.83	26.62
Missing/refused	1.88	
Managerial relations factor		−0.06 (0.98)
Plans to ask for raise		36.10
Age (years)	42.22 (12.53)	42.64 (12.39)
*Race*		
White	62.21	66.45
Black	12.16	11.09
Hispanic	16.96	13.95
Other	8.66	8.51
Female	46.17	48.94
*Occupation*		
Management, business, finance	20.91	25.06
Professional	26.48	25.02
Service	12.40	10.67
Sales	6.50	7.62
Office and administrative support	16.46	17.95
Farming, fishing, forestry	0.38	0.00
Construction and extraction	2.60	2.50
Installation, maintenance, repair	3.07	2.90
Production	4.84	4.71
Transportation	4.97	2.50
Missing/refused	1.38	1.06
*Organization type*		
One of several workplaces	69.75	70.76
Single independent establishment	28.89	28.81
Missing/refused	1.36	0.43
*Organizational size*		
Less than 15 employees	23.01	22.97
15 to 49 employees	20.40	22.07
50 to 99 employees	13.85	13.69
100 to 499 employees	24.27	23.69
500 or more employees	17.09	17.07
Missing/refused	1.38	0.51
*Tenure*		
Less than 1 year	12.70	13.76
1 to less than 2 years	11.75	11.06
2 to less than 5 years	23.89	23.80
5 to less than 10 years	17.84	18.97
10 or more years	33.48	32.28
Missing/refused	0.34	0.15
*N* observations	4,229	2,037

*Note:* Table displays percentages and means (with standard deviations in parentheses) for the variables in our study. Missing or “refused/don’t know” categories are not shown. The restricted sample in column 2 includes those working under a pay secrecy policy (discussion is either discouraged or prohibited). Estimates are weighted by sample weights.

The second column is restricted to workers in organizations with a pay secrecy policy, corresponding to our tests of Hypotheses 4 and 5. Despite the restriction, nearly a third report violating the policy. Compared with the full sample in the first column, workers subject to a pay secrecy policy are overwhelmingly concentrated in private sector for-profit organizations, where unionization rates are lower. In terms of the types of pay secrecy rules these workers are subject to, nearly three-quarters report being discouraged from discussing pay rather than being subject to a formal ban. For these tests of whether pay secrecy rules can be characterized as rules of managerial discretion, we focus on the effects of our two relational variables: a composite standardized scale of managerial relations, and a dichotomous measure of whether the respondent plans to ask for a raise in the next year. Over a third of the sample plans to ask for higher pay. The distributions of other core control variables are broadly similar between the two samples.

## Results

Table 3 presents the results of our analyses of pay talk. Model 1 includes our full range of demographic, occupational, and other organizational controls. In the pooled sample model, the two private sector variables (for-profit and non-profit) are significant and positively signed, consistent with expectations. Limiting the model to Round 1 responses only renders the sector variables nonsignificant, though they are still positively signed. Union membership is unassociated with talking about pay in both versions of the model, contrary to expectations. Consistent with Hypothesis 3, the organizational approaches to pay discussion indicators are significant and negatively signed. Model 2 limits the sample to the 82 percent of respondents that do not have access to pay information as part of their job. Aside from those workers in establishments that publicize pay information, this is the segment of the workforce that must actively seek out wage and salary information should they desire it. The restriction does not alter our organizational relationships of interest, although the effect of the non-profit indicator is no longer significant in the pooled sample version of the model.

**Table 3:** Organizational correlates of violating the salary taboo.

	Model 1		Model 2	
	Full pooled sample	Full round 1 sample	Restricted pooled sample	Restricted round 1 sample
*Sector (ref. = government)*				
Private	0.364^*^	0.276	0.407^*^^*^	0.287
	(0.147)	(0.173)	(0.157)	(0.186)
Non-profit	0.314^*^	0.180	0.265	0.123
	(0.160)	(0.192)	(0.172)	(0.208)
Missing/refused	0.602	0.320	0.570	0.386
	(0.345)	(0.463)	(0.344)	(0.470)
*Union membership (ref. = no)*				
Yes	−0.144	−0.019	−0.179	−0.114
	(0.139)	(0.160)	(0.145)	(0.171)
Missing/refused	−0.815	−0.104	−1.017^*^	−0.903
	(0.480)	(0.798)	(0.490)	(0.802)
*Pay secrecy policy (ref. = discussion permitted)*				
Information is public	−0.348^*^	−0.415^*^	−0.435^*^^*^	−0.422^*^
	(0.142)	(0.167)	(0.153)	(0.179)
Discussion discouraged	−0.471^*^^*^^*^	−0.424^*^^*^^*^	−0.511^*^^*^^*^	−0.390^*^^*^
	(0.105)	(0.129)	(0.115)	(0.140)
Discussion prohibited	−0.790^*^^*^^*^	−0.713^*^^*^^*^	−0.933^*^^*^^*^	−0.804^*^^*^^*^
	(0.144)	(0.177)	(0.153)	(0.191)
Missing/refused	−2.120^*^^*^^*^	−2.641^*^^*^^*^	−2.362^*^^*^^*^	−3.074^*^^*^^*^
	(0.446)	(0.642)	(0.480)	(0.685)
*Occupation (ref. = management, business, finance)*				
Professional	0.422^*^^*^^*^	0.506^*^^*^^*^	0.251	0.280
	(0.126)	(0.142)	(0.141)	(0.161)
Service	0.752^*^^*^^*^	0.827^*^^*^^*^	0.759^*^^*^^*^	0.780^*^^*^^*^
	(0.168)	(0.191)	(0.179)	(0.211)
Sales	0.570^*^^*^	0.485^*^	0.578^*^^*^	0.510
	(0.203)	(0.233)	(0.220)	(0.266)
Office and administrative	0.343^*^	0.421^*^	0.358^*^	0.363
	(0.151)	(0.173)	(0.167)	(0.194)
Farming, fisheries, forestry	0.576	0.566	0.301	0.465
	(0.683)	(0.753)	(0.792)	(0.807)
Construction and extraction	1.047^*^^*^^*^	0.966^*^^*^	0.909^*^^*^	0.671
	(0.302)	(0.361)	(0.338)	(0.399)
Installation, maintenance, repair	0.843^*^^*^	0.785^*^	0.648^*^	0.565
	(0.302)	(0.320)	(0.330)	(0.345)
Production	0.797^*^^*^^*^	0.864^*^^*^^*^	0.676^*^^*^	0.688^*^^*^
	(0.221)	(0.250)	(0.233)	(0.267)
Transportation	0.966^*^^*^^*^	1.291^*^^*^^*^	0.941^*^^*^^*^	1.277^*^^*^^*^
	(0.224)	(0.263)	(0.235)	(0.280)
Missing/refused	−0.473	−0.385	−0.576	−0.529
	(0.424)	(0.477)	(0.449)	(0.498)
*Tenure (ref. = less than 1 year)*				
1 to less than 2 years	0.579^*^^*^^*^	0.470^*^	0.703^*^^*^^*^	0.540^*^^*^
	(0.164)	(0.200)	(0.177)	(0.210)
2 to less than 5 years	0.459^*^^*^	0.464^*^^*^	0.572^*^^*^^*^	0.553^*^^*^
	(0.149)	(0.177)	(0.162)	(0.190)
5 to less than 10 years	0.406^*^	0.403^*^	0.628^*^^*^^*^	0.571^*^^*^
	(0.159)	(0.188)	(0.167)	(0.198)
10 or more years	0.373^*^	0.321	0.635^*^^*^^*^	0.545^*^^*^
	(0.154)	(0.182)	(0.166)	(0.194)
Missing/refused	0.678	0.173	0.520	0.272
	(0.760)	(1.184)	(0.878)	(1.226)
*Organization type (ref. = one of several workplaces)*				
Single independent est.	−0.244^*^	−0.236^*^	−0.214^*^	−0.182
	(0.097)	(0.117)	(0.106)	(0.129)
Missing/refused	0.376	1.190^*^	0.298	1.222^*^
	(0.371)	(0.467)	(0.387)	(0.511)
*Organizational size (ref. = < 15 employees)*				
15 to 49 employees	0.162	0.244	0.144	0.287
	(0.132)	(0.157)	(0.141)	(0.171)
50 to 99 employees	0.105	0.212	0.127	0.259
	(0.142)	(0.174)	(0.152)	(0.191)
100 to 499 employees	0.155	0.178	0.048	0.102
	(0.123)	(0.150)	(0.134)	(0.163)
500 or more employees	0.220	0.194	0.085	0.085
	(0.135)	(0.170)	(0.146)	(0.183)
Missing/refused	0.003	0.274	0.023	0.245
	(0.391)	(0.475)	(0.423)	(0.504)
Age	−0.034^*^^*^^*^	−0.039^*^^*^^*^	−0.036^*^^*^^*^	−0.041^*^^*^^*^
	(0.004)	(0.005)	(0.004)	(0.005)
Female	0.081	0.035	0.160	0.107
	(0.093)	(0.106)	(0.099)	(0.116)
*Race (ref. = White)*				
Black	−0.105	−0.274	−0.156	−0.346
	(0.156)	(0.182)	(0.163)	(0.196)
Hispanic	0.038	−0.273	0.126	−0.191
	(0.131)	(0.151)	(0.139)	(0.161)
Other	−0.069	−0.101	−0.134	−0.210
	(0.168)	(0.183)	(0.175)	(0.197)
Survey round	0.162^*^		0.152	
	(0.072)		(0.080)	
Constant	−0.215	0.223	−0.083	0.418
	(0.301)	(0.331)	(0.326)	(0.361)
*N*	4,229	2,553	3,455	2,095
*R^2^*	0.073	0.082	0.082	0.090
Log likelihood	−2,546.17	−1,507.29	−2,130.89	−1,269.30

*Note:* Table displays log-odds coefficients; standard errors are in parentheses (robust standard errors clustered within individuals are used for pooled sample). Model 2 is limited to workers without access to pay information as part of their jobs. Estimates are weighted by sample weights. Statistical significance indicated by: ^*^P < .05, ^*^^*^P < .01, ^*^^*^^*^P < .001.

Controls associated with pay talk in the two models include certain occupation dummies, tenure on the job, and age. Blue-collar workers in occupations such as manufacturing and production and extraction are more likely to talk about pay than management, business, and finance workers. Compared with the most recent hires (i.e., those with less than a year of experience in their organizations), longer tenured workers are more likely to ask coworkers about wages and salaries. As expected, age is negatively associated with pay talk, while other demographic indicators are not associated with discussing wages and salaries. Interestingly, compared with workers in firms with multiple establishments, those in single establishment firms are less likely to discuss wages and salaries in three of the four models displayed.

**Figure 1:**
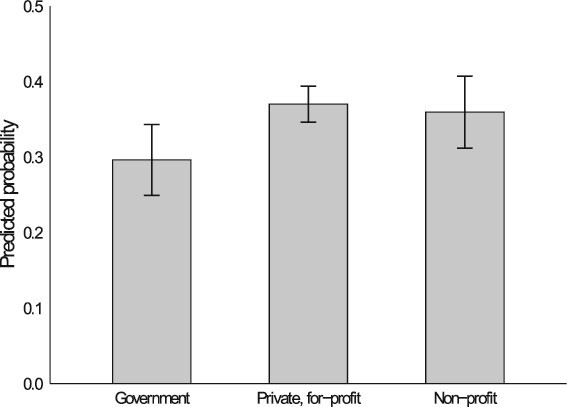
Predicted probabilities that an individual talks to their coworkers about pay, by sector. *Note:* Figure shows the predicted probabilities that an individual talks with their coworkers about pay, by sector. Probabilities are estimated using the results from Model 1 (pooled sample) in Table 3, holding all other controls at their means. “Missing/refused” category is not shown. The bars indicate 95 percent confidence intervals around the estimates.

How large are the effects uncovered in Table 3’s Model 1? Figure 1 displays predicted probabilities based on Model 1’s pooled sample for our first core organizational feature: sector. We generate these probabilities by setting all other covariates at their means. As shown, just over a quarter of workers in public sector establishments discuss pay with colleagues. Compared with private sector establishments, in public sector workplaces managerial control over pay-setting is constrained, and pay scales are often publicized. As a result, according to our predictions, workers in private sector, for-profit establishments are 29 percent more likely and workers in private sector, non-profit establishments are 25 percent more likely to discuss pay than government employees. These results buttress Hypothesis 1.

**Figure 2:**
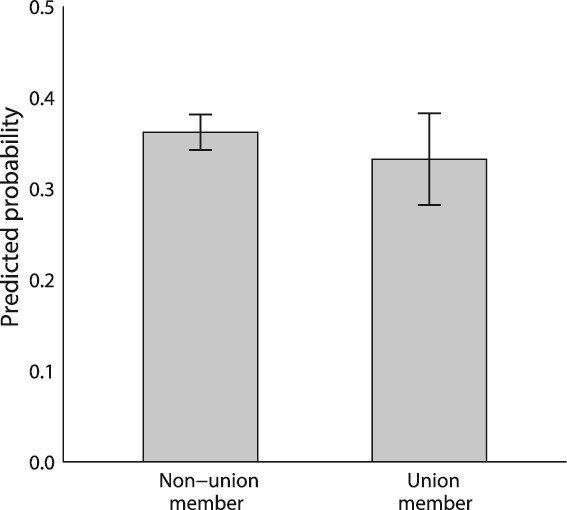
Predicted probabilities that an individual talks to their coworkers about pay, by union membership. *Note:* Figure shows the predicted probabilities that an individual talks with their coworkers about pay, by union membership. Probabilities are estimated using the results from Model 1 (pooled sample) in Table 3, holding all other controls at their means. “Missing/refused” category is not shown. Bars indicate 95 percent confidence intervals around the estimates.

By contrast, we find no evidence that union membership is a significant predictor of talking about pay in any version of our models from Table 3. Figure 2 is again based on Model 1’s pooled sample and displays predicted probabilities of talking about pay for union members and nonmembers. Compared with union members, a slightly smaller percentage of non-members are predicted to discuss pay, but the difference is not significant.

Given the strong correlation between union membership and sector—in our data, the public sector unionization rate is four times that of the private sector rate—the lack of a union effect may stem from the confounding effect of sector. Yet, in supplemental analyses that include sector-by-union interactions, we do not find evidence for within-sector effects. Within the private sector, where pay is rarely public, unionized workers are about 5 percent points less likely discuss pay, but again the difference is not statistically significant. These results appear contrary to Hypothesis 2, but it may also be that our survey is underpowered to detect union differences, especially since union membership is highly correlated with both sector and pay secrecy policy.

**Figure 3:**
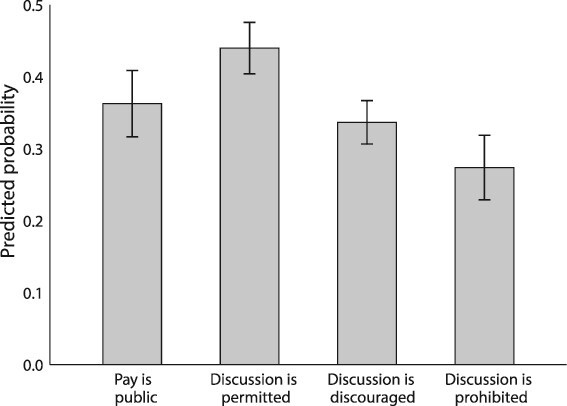
Predicted probabilities that an individual talks to their coworkers about pay, by workplace pay secrecy policy. *Note:* Figure shows the predicted probabilities that an individual talks with their coworkers about pay, by the pay secrecy policy at their workplace. Probabilities are estimated using the results from Model 1 (pooled sample) in Table 3, holding all other controls at their means. “Missing/refused” category is not shown. Bars indicate 95 percent confidence intervals around the estimates.

What about the effects of workplace pay secrecy rules? Figure 3, based on Model 1’s pooled sample from Table 3, displays predicted probabilities of discussing pay by respondents’ workplaces’ approach to pay discussions, and Table 4 tests whether the predicted probabilities between pairs of pay secrecy policies are significantly different from one another. As shown, approximately 36 percent of workers in organizations in which pay is public discuss pay. In the vast majority of organizations, pay is not so transparent. In these workplaces, the predicted probabilities display a sharp downward gradient, consistent with Hypotheses 3a and 3b. Over 40 percent of workers who are allowed to discuss pay in their organizations do so, compared with 34 percent of workers subject to an informal pay secrecy policy, and just 27 percent of workers subject to a formal ban. As Table 4 shows, respondents in workplaces that publicize pay information or discourage or prohibit discussions of pay are significantly less likely to talk with their coworkers about pay than those respondents whose workplaces permit such discussions. Those whose workplaces formally prohibit discussions are significantly less likely to engage in pay talk than those whose workplaces either publicize pay information (by about 9 percent points) or discourage pay discussions (by roughly 5 percent points). While these results indicate that pay secrecy rules matter for suppressing talk about wages and salaries, they also reveal their limitations, with between a third and a quarter of workers in organizations with such rules reporting that they break them.

**Table 4:** Predicted probabilities that an individual talks to their coworkers about pay, by workplace pay secrecy rule.

	Predicted probability	Differences:			
		vs. permitted	vs. public	vs. discouraged	vs. prohibited
Discussion permitted	0.440				
Information is public	0.363	−0.077^*^			
Discussion discouraged	0.337	−0.103^*^^*^^*^	−0.026		
Discussion prohibited	0.274	−0.166^*^^*^^*^	−0.089^*^	−0.063^*^	
Missing/refused	0.096	−0.344^*^^*^^*^	−0.267^*^^*^^*^	−0.241^*^^*^^*^	−0.178^*^^*^^*^

*Note:* Table presents the predicted probabilities that an individual talks with their coworkers about pay, by the pay secrecy rule at their workplace, as well as the pairwise differences between each approach. Probabilities are estimated using the results from Model 1 (pooled sample) in Table 3, holding all other controls at their means. Statistical significance of pairwise differences indicated by: ^*^P < .05, ^*^^*^P < .01, ^*^^*^^*^P < .001.

Part 2 of our investigation focuses on workers subject to pay secrecy rules to test whether relational factors are associated with workers’ violations of them. Table 5 displays the results of logistic regressions limited to respondents who report that they are either discouraged or outright banned from talking about pay. Model 1 includes our organizational and relational factors of theoretical interest as well as our core set of demographic, occupational, and workplace controls; and Model 2 replicates Model 1 but is restricted to workers not granted access to pay information as part of their job.

**Table 5:** Organizational and relational correlates of violating pay secrecy rules, among workers subject to a pay secrecy policy.

	Model 1		Model 2	
	Full pooled sample	Full round 1 sample	Restricted pooled sample	Restricted round 1 sample
*Sector (ref. = government)*				
Private	0.189	−0.214	0.171	−0.194
	(0.324)	(0.404)	(0.353)	(0.433)
Non-profit	0.289	−0.022	0.214	0.020
	(0.349)	(0.434)	(0.378)	(0.466)
Missing/refused	1.132	0.194	1.087	0.185
	(0.595)	(0.666)	(0.624)	(0.685)
*Union membership (ref. = no)*				
Yes	−0.161	−0.124	−0.194	−.188
	(0.340)	(0.398)	(0.358)	(0.422)
Missing/refused	0.387	2.352	−0.370	1.046
	(0.630)	(1.224)	(0.656)	(1.599)
*Pay secrecy policy (ref. = discussion discouraged)*				
Discussion prohibited	−0.433^*^^*^	−0.464^*^^*^	−0.531^*^^*^^*^	−0.597^*^^*^
	(0.140)	(0.180)	(0.149)	(0.196)
Managerial relations index	−0.326^*^^*^^*^	−0.377^*^^*^^*^	−0.265^*^^*^^*^	−0.308^*^^*^^*^
	(0.061)	(0.074)	(0.066)	(0.080)
*Plan to ask for a raise (ref. = No)*				
Yes	0.202	0.074	0.290^*^	0.142
	(0.132)	(0.163)	(0.145)	(0.178)
Missing/refused	−0.281	−1.048	−0.152	−1.101
	(0.572)	(0.744)	(0.615)	(0.784)
*Occupation (ref. = management, business, finance)*				
Professional	0.330	0.515^*^	0.213	0.302
	(0.175)	(0.208)	(0.191)	(0.231)
Service	0.469	0.733^*^	0.529	0.733^*^
	(0.255)	(0.293)	(0.274)	(0.321)
Sales	0.431	0.392	0.469	0.533
	(0.264)	(0.324)	(0.297)	(0.371)
Office and administrative	0.246	0.404	0.216	0.354
	(0.205)	(0.244)	(0.232)	(0.274)
Construction and extraction	0.735	0.780	0.680	0.341
	(0.446)	(0.594)	(0.532)	(0.693)
Installation, maintenance, repair	0.767^*^	0.768	0.702	0.675
	(0.377)	(0.465)	(0.420)	(0.512)
Production	0.632^*^	0.661	0.609	0.572
	(0.306)	(0.382)	(0.319)	(0.397)
Transportation	1.998^*^^*^^*^	2.699^*^^*^^*^	1.904^*^^*^^*^	2.570^*^^*^^*^
	(0.379)	(0.475)	(0.394)	(0.486)
Missing/refused	−0.598	−0.752	−0.926	0.416
	(0.793)	(0.753)	(0.796)	(0.740)
*Tenure (ref. = less than 1 year)*				
1 to less than 2 years	0.496^*^	0.585	0.697^*^^*^	0.734^*^
	(0.237)	(0.302)	(0.263)	(0.324)
2 to less than 5 years	0.332	0.497	0.502^*^	0.701^*^
	(0.221)	(0.275)	(0.241)	(0.298)
5 to less than 10 years	0.599^*^	0.701^*^	0.902^*^^*^^*^	1.017^*^^*^^*^
	(0.234)	(0.283)	(0.250)	(0.301)
10 or more years	0.598^*^^*^	0.727^*^^*^	0.999^*^^*^^*^	1.075^*^^*^^*^
	(0.225)	(0.278)	(0.247)	(0.303)
Missing/refused	−0.862		−0.700	
	(1.376)		(1.374)	
*Organization type (ref. = one of several workplaces)*				
Single independent est.	−0.291^*^	−0.250	−0.291	−0.225
	(0.142)	(0.180)	(0.158)	(0.197)
Missing/refused	−0.759	0.352	−0.791	0.367
	(0.836)	(1.037)	(0.837)	(1.086)
*Organizational size (ref. = < 15 employees)*				
15 to 49 employees	0.382^*^	0.266	0.438^*^	0.429
	(0.184)	(0.233)	(0.196)	(0.250)
50 to 99 employees	0.373	0.251	0.422	0.315
	(0.203)	(0.262)	(0.219)	(0.292)
100 to 499 employees	0.285	0.226	0.241	0.161
	(0.178)	(0.223)	(0.194)	(0.240)
500 or more employees	0.254	0.234	0.141	0.128
	(0.191)	(0.248)	(0.207)	(0.266)
Missing/refused	0.311	−0.088	0.388	−0.272
	(0.713)	(0.972)	(0.738)	(0.899)
Age	−0.037^*^^*^^*^	−0.048^*^^*^^*^	−0.040^*^^*^^*^	−.055^*^^*^^*^
	(0.006)	(0.007)	(0.006)	(0.008)
Female	0.028	−0.103	0.179	0.042
	(0.136)	(0.167)	(0.150)	(0.180)
*Race (ref. = White)*				
Black	0.144	0.174	0.050	0.006
	(0.222)	(0.254)	(0.234)	(0.267)
Hispanic	0.061	−0.131	0.036	−0.146
	(0.201)	(0.241)	(0.222)	(0.258)
Other	0.013	0.001	−0.102	−0.111
	(0.243)	(0.270)	(0.237)	(280)
Survey round	0.126		0.045	
	(0.109)		(0.119)	
Constant	−0.566	0.344	−0.425	0.556
	(0.500)	(0.598)	(0.546)	(0.644)
*N*	2037	1207	1648	973
*R^2^*	0.079	0.108	0.086	0.115
Log likelihood	−1166.87	−673.44	−979.87	−556.91

*Note:* Table displays log-odds coefficients; standard errors are in parentheses (robust standard errors clustered within individuals are used for pooled sample). All models in this table include those working under a pay secrecy policy (discussion is either discouraged or prohibited), and Model 2 is further limited to workers without access to pay information as part of their jobs. Estimates are weighted by sample weights. Statistical significance indicated by: ^*^P < .05, ^*^^*^P < .01, ^*^^*^^*^P < .001.

In terms of organizational factors, as shown in the first model, restricting the sample to workers subject to a pay secrecy rule renders sector nonsignificant. This is unsurprising given the strong correlation between sector and secrecy policy: just 10 percent of government employees report being subject to a pay secrecy rule compared with nearly two-thirds of for-profit, private sector workers. Union membership is also unassociated with discussing pay. The type of pay secrecy rule matters: workers who are formally barred from discussing wages and salaries are significantly less likely to break this rule compared with workers whose managers actively discourage them from talking about pay.

Model 1 provides evidence for Hypothesis 4 and partial evidence for Hypothesis 5. Positive relations with management, proxied by our managerial relations scale, lower the likelihood a worker will break a pay secrecy rule, consistent with Hypothesis 4. A worker planning to ask for a raise is not significantly more likely to defy her workplace’s pay secrecy rule, but the coefficients are positively signed in both the pooled and Round 1 samples. Moving to Model 2, this relational effect is significant in the pooled sample version of the model that includes all controls and excludes workers who receive pay information as part of their jobs. Across all specifications, the managerial relations scale is significantly associated with disobeying pay secrecy rules. Other controls operate mostly as expected, although with far fewer significant effects than in Table 3, likely due in part to the reduced sample size.

**Figure 4:**
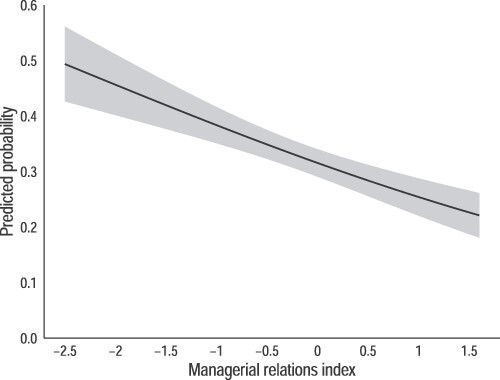
Predicted probabilities that an individual talks to their coworkers about pay, by managerial relations index. *Note:* Figure shows the predicted probabilities that an individual talks with their coworkers about pay, by the managerial relations index. Probabilities are estimated using the results from Model 1 (pooled sample) in Table 5, holding all other controls at their means. Shaded area indicates 95 percent confidence interval around the estimates.

**Figure 5:**
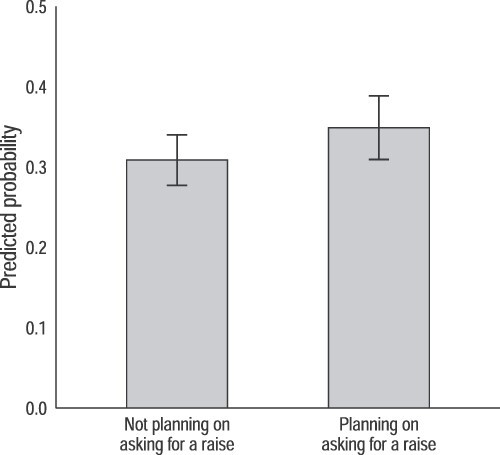
Predicted probabilities that an individual talks to their coworkers about pay, by whether they plan on asking for a raise. *Note:* Figure shows the predicted probabilities that an individual talks with their coworkers about pay, by whether or not they plan on asking for a raise. Probabilities are estimated using the results from Model 1 (pooled sample) in Table 5, holding all other controls at their means. “Missing/refused” category is not shown. Bars indicate 95 percent confidence intervals around the estimates.

How large are these relational effects? In figures 4 and 5, we display workers’ predicted probabilities of defying pay secrecy rules based on Model 1’s pooled sample version by their location on our managerial relations index (fig. 4) and by whether they plan to ask for a raise in the next year (fig. 5). The downward gradient evident in figure 4 reveals how the probability of breaking pay secrecy rules declines as managerial relations within the workplace improves. At the tenth percentile of the managerial relations distribution, over 40 percent of workers are predicted to violate their organization’s pay secrecy rule. At the ninetieth percentile, less than a quarter are.

The effect of planning to ask for a raise is not as large, but still reveals the possible relationship between this particular workplace relation and the likelihood a worker will violate a rule against discussing pay. As shown in the figure, a third of workers who do plan on asking for a pay increase say they discuss pay with colleagues compared with 29 percent of workers with no plans to ask for a raise. In this specification—Model 1’s pooled sample from Table 5—the difference is not significant. But in the second model of the pooled sample restricted to workers not privy to pay information as a condition of their jobs, workers planning to ask for a raise are 8 percent points more likely to break their organization’s pay secrecy rule—a significant difference.

## Discussion and conclusion

In this investigation, we uncover the set of organizational and relational factors that structure pay talk within workplaces. We theorize that the likelihood a worker will discuss pay varies according to whether pay information is scarce, and to the extent employers control pay-setting. Under these conditions—uneven distribution of pay information and employer discretion in allocating pay—a worker’s knowledge of pay scales can shift bargaining dynamics in the worker’s favor. The first part of our analyses confirms that organizational factors are related to the frequency of pay talk. In particular, organizational rules against discussing pay stand out as consistently associated with a worker’s willingness to discuss wages and salaries with her colleagues.

Yet, similar to other analyses of workplace rule-breaking (Martin et al. 2013), the second part of the investigation indicates that violations of these rules are common. These violations are relationally patterned: less frequent among workers who report positive managerial relations within their organization, and possibly more frequent among workers who plan to ask for a raise in the coming year. Rules of managerial discretion widen managerial autonomy by restricting worker autonomy; disregarding these rules can arm workers with important information in relational claims-making (Avent-Holt and Tomaskovic-Devey 2014). The impetus to gain access to such information increases as trust in one’s superiors decreases, and at important junctures in one’s career—such as a salary renegotiation.

Aside from our organizational and relational correlates of interest, the findings also hint at the possibility of changing relationships between the workplace and younger generations of workers. Consistent throughout the analyses is the robust, negative relationship between age and discussing pay with co-workers, even in workplaces that maintain pay secrecy rules. The willingness of younger workers to break with broadly held norms and workplace rules may signal an emerging erosion of the legitimating frames (Roscigno 2011) many workplaces rely on to uphold existing power relations. But it also could be that eventually these workers will grow more reluctant to discuss pay as their careers progress. Adjusting for work experience lends confidence to our preliminary conclusion that the breakdown of the mid-to-late twentieth century “normative employment contract” (Rubin 2012, p. 33) has eroded support for maintaining pay secrecy among younger generations; yet, a decisive answer to this question awaits panel data spanning many years that include information about pay transparency and worker attitudes toward the salary taboo.

Other shortcomings remain. Restricting results to Round 1 survey responses only renders certain findings nonsignificant. However, the directionality of the coefficients remains consistent, lending confidence that the differences across samples reflect a lack of power in the Round 1 models more than anything substantive. Only surveys with larger samples that span a longer time span can adjudicate whether the sector and raise effects we find in our pooled sample specifications are robust.

It is also possible that self-reporting bias results in workers underreporting whether they engage in pay talk. That is, workers subject to a pay secrecy rule may hesitate to admit that they talk with their coworkers about pay, biasing our rates of pay secrecy rule violations downward. Our survey relies on employees’ self-reports, and therefore we cannot validate responses to check for such biases. At the same time, our data suggest that this possible source of bias may not be extensive. For self-reporting bias to strongly influence our results, a substantial share of workers subject to a pay secrecy rule would have to be more comfortable breaking this rule and risking sanction at their place of employment than admitting the violation on an anonymous survey. We cannot think of a reason why this would be the case.

Two surprising findings warrant further investigation. First, we hypothesized that union membership would relate to pay talk; results indicate otherwise. We argue that the impersonal factors underlying many union-negotiated pay structures—seniority and job title, in particular—render pay information predictable and less potentially powerful, given that workers who know a colleague’s job and tenure at the organization have a good sense of her earnings. Yet, the protections unions provide workers may simultaneously lower fears of discussing pay around colleagues, and these two counteracting forces may result in the nonsignificant union membership coefficients. Or it may be that our survey is underpowered, given the strong correlations between sector, unionization, and pay secrecy rules.

Second, we did not anticipate that type of organization would be consistently related to discussing pay. Models reveal that workers in single establishments are significantly less likely to discuss wages and salaries compared with workers in multi-establishment firms. Other organizational factors not already included in our models that distinguish single establishments from those that are part of a larger firm remain unknown. It could also be that organizational cultures of single-establishment firms differ from those of multi-establishment enterprises. Future research is needed to uncover the mechanisms explaining this surprising result.

While the survey contains a set of managerial relations measures, it lacks a corresponding set of variables capturing workers’ relationships with their peers—their horizontal workplace relations. We view the worker tenure measure as a proxy for horizontal relations, reasoning that longer tenured workers are more likely to have established the close networks with peers through which pay information flows. But future analyses of the salary taboo should leverage data with direct measures of workers’ trust in and support of their co-workers.

Our survey is unique in that it contains information on worker and employer approaches to pay secrecy. But we survey individuals about themselves and their workplaces, and thus lack the rich set of organizational measures that often come with organizational-level data. As a result, we rely on a set of proxy measures to test whether our organizational measures are associated with pay talk. To our knowledge, with the exception of Cullen and Perez-Truglia’s (2022, 2023) case studies, no organizational-level dataset includes information on pay discussions and pay secrecy rules. Future data collection efforts—ideally the creation of large-scale, matched employer-employee datasets—would enable researchers to include a broader set of organizational characteristics in analyses of pay information flows within workplaces, and provide sharper tests of our core findings in this investigation.

Organizational-level data that span years would also allow researchers to uncover the specific effects of pay secrecy rules on workers’ pay. We conceptualize pay secrecy rules as rules of managerial discretion, similar to non-compete agreements, another workplace rule that expands managerial prerogatives by reducing worker power. Research on the effects of non-competes is clear: they result in lower pay and less job mobility for workers subject to them (Starr, Prescott, and Bishara 2020; Starr, Prescott, and Bishara 2021). What are the broader effects of pay secrecy rules? Existing research provides mixed results, often depending on the outcome under investigation (e.g., Cullen and Pakzad-Hurson 2023; Frey 2021). This emerging literature focuses largely on the effects of national or subnational pay transparency policies on gender wage gaps or overall worker pay. We argue that pay secrecy rules grant employers greater control over pay-setting, and thus we would expect greater pay variation in those organizations where these rules are in place. Auxiliary analyses (available upon request) modeling inequality in log hourly earnings by pay secrecy policy suggest pay variation is highest among workers subject to formal prohibitions against pay talk, at least among college-educated workers. But a lack of a full set of relevant organizational-level controls prevents us from adjusting for factors correlated with pay secrecy rules and pay outcomes that could be driving the correlation. Nor can we rule out selection effects: workers who desire highly differentiated pay may select into workplaces with strictures against discussing wages and salaries. Organizational-level data, especially data that span changes in pay secrecy policies over time, could help establish whether the wider variation we uncover is reflective of worker desires or simply stems from the greater managerial discretion pay secrecy offers.

Shortcomings aside, this article provides the most comprehensive investigation into pay talk to date. Unlike previous accounts, our findings suggest that the reluctance to discuss pay is far from universal, and that factors other than a worker’s age help explain whether workers will talk about pay. Similar to pay negotiations (Sauer et al. 2021), we find that the willingness to discuss pay is not simply an individual-level phenomenon, but is patterned by key organizational and relational characteristics that help us understand who is likely to keep pay information secret, and who is likely to share it. Whether or not a workplace has a pay secrecy rule in place is one characteristic that clearly influences the salary taboo. These pay secrecy rules are both common and, in many situations, themselves a violation of the rules: courts have consistently found that organizational rules against discussing wages and salaries violate the National Labor Relations Act (Gely and Bierman 2003). Here again, we see a parallel with non-competes in that a growing number of states and, recently, the Federal Trade Commission have ruled non-competes unenforceable in most contexts. The ubiquity of both of these rules of discretion speaks to the disconnect between workplace and legal governance, and call for further research to explore the underpinnings and implications of a set of workplace laws that many employers ignore. Their prevalence across the non-union, private sector landscape also suggests the limitations of the emerging set of state-level laws meant to combat them. Outreach and enforcement must be paired with the passage of anti-secrecy laws if they are to work as intended. Moreover, even in those workplaces that allow pay discussions, most workers do not discuss pay, suggesting that informal norms inhibit many workers from uncovering information potentially relevant to their wage or salary. Public outreach about the potential benefits of salary transparency paired with powerful legal remedies could help shift this long-standing workplace custom.

The data we rely on for this study predate the workplace disruptions caused by COVID-19. How the pandemic affects the key factors that structure pay talk remains to be seen. For example, the growth of telework may weaken the horizontal relations important for sharing sensitive information. At the same time, eroding managerial relations between employers and workers who were dissatisfied with their organizations’ protections and flexibility during the pandemic may increase the sharing of pay information.

Pay information is not neutral, but potentially powerful, and thus trends in workplace pay transparency alert us to shifting power dynamics in contemporary organizations. This study provides a blueprint for understanding the key factors that support salary secrecy in contemporary organizations, as well as those that may be weakening its hold.

## Supplementary Material

sf-may-23-245-File002_soae130

## Data Availability

The data underlying this article will be made available in the Open Science Framework at https://osf.io/jwu7k/?view_only=57189a0e7fe14a619a65806acec09fbd.

## Footnotes

1 See https://www.dol.gov/agencies/wb/equal-pay-protections for an updated list of policies against pay secrecy rules in workplaces.

2 We thank an anonymous reviewer for suggesting this formulation.

3 Unlike Luekemann and Abendroth’s (2018) understanding of career advancement discussions, we do not view all instances of pay talk as claims-making over organizational revenue. Pay discussions can reveal one worker is overpaid relative to a similarly situated other. For the overpaid worker, this revelation is unlikely to activate a claim on organizational resources, at least in the short term. However, the new information can augment the worker’s leverage vis-à-vis her employer by granting the worker knowledge of the employers’ pay-setting practices, which could be used in future rounds of claims making.

4 One of the original intentions of the multiple waves of data collection was to track the effect of state-level policy changes, particularly in Massachusetts, which passed new legislation related to pay transparency in the time between the two rounds of data collection. That analysis is beyond the scope of the present investigation.

5 Descriptives in Table 1 are consistent when restricting the sample to individuals’ responses from Round 1 of the survey only. For example, the share of workers who report that they know the wages or salaries of at least some of their coworkers is nearly identical whether we include all observations from both survey rounds (63.76 percent, as in Table 1) or just those respondents from Round 1 (63.15 percent). Similarly, among those who know their coworkers’ pay, 55.86 percent of the full sample and 54.59 percent of the Round 1 sample know from talking with their coworkers.

6 As in Table 1, descriptives remain similar when analyzing Round 1 survey responses only. For example, similar shares of the pooled and Round 1 samples report talking with their coworkers about pay (35.62 and 34.47 percent, respectively), working in the private sector (67.32 and 66.94 percent), belonging to a union (13.72 and 13.69 percent), and that they are either discouraged or prohibited from talking about pay with co-workers (48.46 and 48.59 percent).

## About the authors

**Patrick Denice** is an Associate Professor of Sociology at the University of Western Ontario. His research examines the implications of workplace policies and institutions for workers’ wages, non-traditional and extended pathways to and through postsecondary education, and how public school choice policies shape patterns of racial/ethnic segregation and stratification within and across schools. Some of his recent works have been published in *Sociology of Education*, *Demography*, and *Social Science Research*.

## Notes

**Jake Rosenfeld** is a Professor of Sociology and Resident Fellow of the Weidenbaum Center on the Economy, Government, and Public Policy at Washington University in St. Louis. His research focuses on labor market inequality and workplace dynamics in the US and other advanced economies. He has published two books with Harvard University Press on these topics, along with journal articles in the *American Sociological Review*, *American Journal of Sociology*, and *Social Forces*, among other outlets.

## Notes

**Shengwei Sun** is a Senior Research Analyst at the National Women’s Law Center, where she leads research on income security and childcare policies and ensures rigor in data analysis. Shengwei was previously a senior research associate at the Institute for Women’s Policy Research and a postdoctoral fellow at the Washington University in St. Louis. She holds a PhD in Sociology from the University of Maryland, College Park.
