# Supplementary material for: Pay talk in contemporary workplaces
Source: Soc Forces. 2024 Sep 9;103(3):839–64. doi: 10.1093/sf/soae130 (PMC11726808; doi:10.1093/sf/soae130)
Supplement: sf-may-23-245-File002_soae130 [file sf-may-23-245-file002_soae130.pdf]

PAY TALK IN CONTEMPORARY WORKPLACES
Supplemental Material

Construction of the managerial relations index

Table A1: Factor loadings and Cronbach's alpha for the managerial relations index

Item	Factor loadings
How good are managers at:	
Seeking out employees' views	0.886
Responding to employees' suggestions	0.896
Allowing employees to influence decisions	0.863
To what extent do you agree that managers:	
Deal with employees honestly	0.892
Treat employees fairly	0.881
Keep their promises	0.898
Cronbach's alpha for 6-item index	0.945

Notes: For the first 3 questions, respondents answered on a scale from 1=very poor to 5=very good; for the second 3 questions, respondents answered on a scale from 1=strongly disagree to 5=strongly agree.

Supplemental Analyses and Robustness Checks

Overall, we find strong empirical support for Hypotheses 3 and 4, and partial support for Hypotheses 1 and 5. Numerous robustness checks and alternative specifications lend confidence to these core findings. Models that include respondents' own pay (i.e., one's hourly wage or logged hourly wage) or their perceived pay rank (a question asking respondents to estimate where their wages or salary ranks compared to their coworkers doing a job similar to or the same as theirs at their workplace, on a 5-category scale ranging from "top 10%" to "bottom 10%") do not change the core results. Models that replace our 11-category occupation measure with an expanded 23-category measure produce substantively similar results, as do models that adjust for respondents' region or educational attainment.

A potential concern about our finding of a relationship between poor managerial relations and discussing pay is reverse causality: workers who talk about pay with colleagues may

perceive poorer managerial relations due to the wage disparities they discover. Supplemental tests (available upon request) adjusting for pay satisfaction produce similar results.

We find no main effects in Tables 3 and 5 for race/ethnicity or gender. But given the significance of occupation in our models, along with the persistence of gender and racial/ethnic segregation across occupations, in other supplementary analyses we include a full battery of occupational composition measures to see if respondents' willingness to discuss pay and to violate pay secrecy policies varies according to the gender and racial/ethnic composition of their occupation. In other words, occupations may matter for whether workers share pay information because of the occupational or class structure itself (as tested by the inclusion of occupational controls in Tables 3 and 5) or because of occupational sorting. These measures include the percentage of each detailed occupation (23-category) that is female, non-Hispanic white, non-Hispanic African American, Hispanic, and other, and two measures of occupational segregation. The first segregation measure is a set of dissimilarity indices capturing the extent of segregation by gender and race/ethnicity within the 23 detailed occupations. The second is a measure adapted from Charles and Grusky (1995; see also Charles and Bradley 2009) that indicates how over- or under-represented women (or non-Hispanic African-Americans, Hispanics, and those of other racial/ethnic groups) are in a given detailed occupation relative to the average occupation.[1] The inclusion of these compositional and segregation measures do not alter our core findings, and they are generally not significantly related to either discussing pay or breaking pay secrecy rules.

[1] These occupation measures are based on the 2014-2018 American Community Survey (ACS) 5-year estimates, not our original survey data. The sample used to create these measures consists of employed persons who work at least 30 hours per week, are 18-81 years of age, and are neither self-employed nor in the military.

References

Charles, Maria and Karen Bradley. 2009. “Indulging Our Gendered Selves? Sex Segregation by Field of Study in 44 Countries.” *American Journal of Sociology* 114:924–976.

Charles, Maria and David B. Grusky. 1995. “Models for Describing the Underlying Structure of Sex Segregation.” *American Journal of Sociology* 100:931–971.
